# GGV formula attenuates CCl_4_-induced hepatic injury in mice by modulating the gut microbiota and metabolites

**DOI:** 10.3389/fnut.2025.1564177

**Published:** 2025-05-27

**Authors:** Yifang Wang, Yihua Zhang, Yinting He, Shixun Zhang, Bo Huang, Yarong Wang, Weibin Lin, Weicheng Xiao, Zhenzhuang Zou, Guozhen Cui

**Affiliations:** ^1^School of Bioengineering, Zhuhai Campus of Zunyi Medical University, Zhuhai, China; ^2^Department of Pediatrics, The Fifth Affiliated Hospital of Zunyi Medical University, Zunyi, China; ^3^Zhuhai Jinwan Biomedical Industry Research Institute, Zhuhai, China; ^4^Department of Pediatrics, Shenzhen Futian District Maternity and Child Health Care Hospital, Shenzhen, China

**Keywords:** hepatoprotective effect, glutathione, *Ganoderma lucidum* extract, vitamin C, gut microbiota, metabolomics

## Abstract

**Background:**

Liver disease is a global health issue requiring effective therapeutic interventions. Although the individual hepatoprotective properties of glutathione, *Ganoderma lucidum* extract, and vitamin C are well-documented, their combined effects remain to be elucidated.

**Objective:**

This study aims to investigate the hepatoprotective potential of a functional food formula named GGV to mitigate acute liver injury induced in mice.

**Methods:**

GGV was orally administered in a mouse model of carbon tetrachloride (CCl_4_)-induced acute liver injury. Liver function was assessed by measuring serum and hepatic biomarkers. Gut microbiota composition and diversity were evaluated using 16S rRNA gene sequencing. Serum metabolomic profiling was conducted using UPLC-Q/TOF-MS.

**Results and conclusion:**

GGV administration significantly ameliorated CCl_4_-induced liver dysfunction, exhibiting greater efficacy than its individual components. Gut microbiota analysis revealed that GGV treatment restored the microbial diversity and composition disrupted by CCl_4_ exposure. Metabolomic profiling further indicated that GGV normalized phospholipid, fatty acid, and bile acid levels. Correlation analysis identified specific microbial genera associated with serum bile acid profiles, suggesting that the hepatoprotective effects of GGV are mediated through modulation of gut microbiota composition and metabolites. Taken together, these findings support the potential of GGV as a promising dietary intervention for promoting liver health through the liver-microbiota-gut axis.

## 1 Introduction

The liver plays an important role in metabolic regulation, detoxification, and immune responses, making it highly susceptible to injury from external agents such as pharmaceuticals and environmental toxins. This susceptibility poses a serious threat to human health, contributing to approximately 2 million deaths annually, accounting for 4% of global mortality (1). Diseases such as viral hepatitis (hepatitis B and C), nonalcoholic fatty liver disease (NAFLD), alcoholic liver disease (ALD), and drug-induced liver injury (DILI) substantially contribute to this burden, posing a major public health challenge (2). With the aging population and the increasing prevalence of metabolic diseases, the incidence of liver-related diseases is expected to increase in the coming years. To better understand the underlying mechanisms of liver disease and explore therapeutic strategies, researchers often use animal models. The pathophysiology of acute liver injury is complex and involves various etiologies, including viral infections, drug toxicity, and ischemia. Among these models, the carbon tetrachloride (CCl_4_)-induced acute liver injury model is widely used because it effectively mimics human liver conditions, such as acute liver injury, fibrosis, and oxidative stress (3). This model is invaluable for studying the pathophysiology of liver damage and evaluating potential hepatoprotective agents (4).

Current treatments for liver diseases include antioxidants, hepatoprotective agents, anti-inflammatory drugs, and antivirals, all aimed at preventing or mitigating liver damage (5). For example, silymarin, an antihepatotoxic agent, is a widely accepted drug for the treatment of liver diseases (6). It has also demonstrated antioxidant, hypolipidemic, and hypoglycemic effects, extending its potential therapeutic applications to cardiovascular health (7). However, the clinical use of silymarin is limited by factors such as drug interactions and pharmacokinetic variability, which can significantly affect its effectiveness and lead to inconsistent therapeutic outcomes (8, 9). Furthermore, although some studies report improvements in liver function markers, there is no conclusive evidence that silymarin significantly enhances survival rates in severe liver conditions such as cirrhosis (10). Adverse effects, including gastrointestinal disturbances and allergic reactions, have also been observed in certain individuals (11). These challenges underscore the necessity for more effective and safer hepatoprotective therapies.

Glutathione, a key molecule involved in maintaining cellular redox homeostasis, has shown promise in protecting the liver from oxidative damage in various liver disorders. S-acetylglutathione (SAG), a prodrug of glutathione, has been found to mitigate CCl_4_-induced liver toxicity by restoring oxidative balance, promoting mitophagy, and reducing inflammation (12). Similarly, vitamin C, known for its antioxidant, anti-inflammatory, and antifibrotic properties, has exhibited hepatoprotective effects in liver conditions such as ALD, NAFLD, DILI, and viral hepatitis. In CCl_4_-induced liver injury, vitamin C supplementation has been shown to improve clinical outcomes and reduce hepatomegaly by restoring metabolic balance and reducing inflammation (13). Additionally, *Ganoderma lucidum* extract has been reported to neutralize free radicals and reduce oxidative stress in the liver, with its triterpenoids and polysaccharides providing protection against CCl_4_-induced liver damage with minimal toxicity (14).

Despite the well-documented individual benefits of glutathione, *Ganoderma lucidum* extract, and vitamin C, their combined hepatoprotective potential remains unexplored. This study aims to address this gap by evaluating the enhanced liver-protective effects of GGV, an innovative therapeutic formulation combining these 3 compounds. Using a CCl_4_-induced acute liver injury model in C57BL/6J mice, we investigated the hepatoprotective efficacy of GGV through comprehensive analyses of gut microbiota composition and serum metabolome.

## 2 Materials and methods

### 2.1 Materials and reagents

Glutathione (CAS: 70-18-8) was obtained from Yunnan Jida Biotechnology Co., Ltd. (Yunnan, China). The aqueous extract of *Ganoderma lucidum* (Gan) was purchased from Wuhu Acegem Biotechnology Co., Ltd. (Anhui, China). Vitamin C (CAS: 50-81-7) was purchased from Heilongjiang NHU Biotechnology Co., Ltd. (Heilongjiang, China). Silymarin and CCl_4_ were obtained from Aladdin Biochemical Technology Co. Ltd. (Shanghai, China). Biochemical assay kits for alanine aminotransferase (ALT), aspartate aminotransferase (AST), total cholesterol (TC), triglyceride (TG), and superoxide dismutase (SOD) were purchased from Nanjing Jiancheng Bioengineering Institute (Nanjing, China).

### 2.2 Animals and treatment

Male C57BL/6J mice, aged 6–8 weeks and weighing 18–20 g, were obtained from Zhuhai BesTest Bio-Tech Co., Ltd. (Zhuhai, China). The animals were housed in the animal facility of Zunyi Medical University under controlled conditions: 23 ± 2°C, 35% relative humidity, and a 12-h light/dark cycle. The mice were provided ad libitum access to water and food as previously described (15). The experimental protocols were approved by the Animal Welfare Ethics Committee of Zunyi Medical University (Approval No: ZHSC-2-[2024]023). The human-recommended daily doses for the glutathione-based formula components are as follows: 100 mg/day of glutathione, 400 mg/day of *Ganoderma lucidum* extract (equivalent to 4 g of raw material), and 400 mg/day of vitamin C, yielding a total dose of 900 mg/day. Silymarin was administered at 300 mg/day. The human doses were converted to mouse-equivalent doses using a standard adult human body weight of 60 kg, resulting in the following dosages: 0.00166 g/kg/day for glutathione, 0.00666 g/kg/day for *Ganoderma lucidum* extract, and 0.00666 g/kg/day for vitamin C. The combined dose of the formula was 0.015 g/kg/day, while silymarin dose was 0.15 g/kg/day. For the low- and high-dose treatment groups, 10-fold and 30-fold the human-equivalent doses were administered, respectively.

The mice were randomly divided into eight groups: control, vehicle, positive control (0.15 g/kg silymarin), glutathione (0.0166 g/kg), *Ganoderma lucidum* (Gan, 0.0666 g/kg), vitamin C (0.0666 g/kg), and GGV groups at two doses (0.15 g/kg, 0.45 g/kg). The control and model groups received distilled water. For the positive control group, silymarin was dissolved in 0.5% sodium carboxymethyl cellulose (Shanghai Shenguang Edible Chemicals Co., Ltd., Shanghai, China) as previously described (16). Each compound was administered daily by oral gavage at a volume of 0.1 mL per 10 g of body weight for 30 consecutive days. Body weight was recorded every 5 days, and dosages were adjusted accordingly.

To induce acute liver injury, CCl_4_ was diluted to 1% in peanut oil and administered via oral gavage at a dose of 5 mL/kg, which is equivalent to 80 mg/kg CCl_4_. On day 30, following a 16-h fasting period, all the mice except those in the control group received a single dose of CCl_4_. The control and vehicle groups were administered only peanut oil. The treatment groups continued receiving their respective compounds, with a minimum 4-h interval between CCl_4_ administration and the test compounds. 24 h after treatment with CCl_4_, the mice were euthanized, and blood, liver, and fecal samples were collected for further analysis.

### 2.3 Biochemical analysis of the serum and liver of the mice

Serum samples were thawed on ice and analyzed for AST, ALT, TG, and TC levels using commercially available assay kits following the manufacturers’ instructions. For liver tissue analysis, 0.1 g of liver tissue was homogenized in 0.9 mL of saline solution and centrifuged at 1,600 × g for 10 min at 4°C. The supernatant was used to measure protein concentration and SOD activity according to the corresponding kit protocol.

### 2.4 H&E Staining of mouse liver tissue

The left lobe of the mouse liver was fixed in 4% paraformaldehyde for 24 h at room temperature. After fixation, the tissue was sectioned into slices, dehydrated, embedded in paraffin, and stained with hematoxylin and eosin (H&E). Pathological changes in the liver tissue were examined under a light microscope (Olympus BX 50, Tokyo, Japan). Liver pathology was evaluated using the pathological scoring system as previously described (17).

### 2.5 Analysis of the gut microbiota in mice

The gut microbiota composition of the mice was analyzed using 16S rRNA sequencing as previously described (18). Genomic DNA was extracted from fecal samples using a commercial extraction kit. The purity and concentration of the extracted DNA were measured to ensure high quality. The V3–V4 region of the 16S rRNA gene was amplified by polymerase chain reaction (PCR). PCR products were quantified, and library preparation was conducted according to the standard protocol for the NEBNext^®^ Ultra^™^ II DNA Library Prep Kit for Illumina^®^ (New England Biolabs, United States). Paired-end sequencing (PE 250) was then performed using the Illumina NovaSeq 6000 platform (Guangdong Magigene Biotechnology Co., Ltd., Guangzhou, China). Sequences with ≥ 97% similarity were clustered into operational taxonomic units (OTUs) using USEARCH software. Taxonomy was assigned based on the SILVA database.

### 2.6 Microbial bioinformatics analysis

Microbial bioinformatics analysis was performed as previously described (19). Briefly, beta diversity was analyzed using principal coordinate analysis (PCoA) to compare the microbial communities between the groups. Differential abundance analysis was conducted to identify specific taxa with significant variation across groups. The linear discriminant analysis effect size method was used to detect potential microbial biomarkers. Statistical significance for multiple group comparisons was assessed using the nonparametric Kruskal-Wallis rank sum test to detect species with significant differences in abundance, followed by pairwise comparisons using Wilcoxon rank sum tests.

### 2.7 Preparation of serum samples for mass spectrometry analysis

Serum samples were thawed on ice, and 100 μL of each sample was mixed with 300 μL of precooled methanol and acetonitrile (2:1, v/v). The mixture was vortexed for 1 min to precipitate the proteins. The samples were then centrifuged at 4,000 × g for 20 min at 4°C. The supernatant was collected and filtered through a 0.22 μm micropore filter before analysis. Ultrahigh-performance liquid chromatography coupled with quadrupole time-of-flight mass spectrometry (UHPLC-Q-TOF/MS, Waters Corp., Milford, United States) was used for sample analysis.

### 2.8 Mass spectrometry conditions and analysis

Mass spectrometry conditions and data analysis were performed as previously described (19, 20). Chromatographic separation was carried out using a Waters Acquity™ UPLC system (Waters Corp., Milford, United States) equipped with an ACQUITY UPLC BEH C18 column (2.1 mm × 50 mm, 1.8 μm) maintained at 40°C. The mobile phases consisted of 0.1% formic acid in water (solvent A) and acetonitrile (solvent B). The elution followed a linear gradient at a flow rate of 0.3 mL/min under the following conditions: 5–20% B from 0 to 1 min, 20–25% B from 1 to 5.5 min, 25–30% B from 5.5 to 6 min, 30% B from 6 to 8 min, 30–35% B from 8 to 9 min, 35–65% B from 9 to 17 min, 65–100% B from 17 to 18 min, 100% B from 18 to 19 min, and 5% B from 19.1 to 20 min. The injection volume was set at 2 μL per sample.

Mass spectrometric analysis was performed using a Waters SYNAPT XS system (Waters Corp., Milford, United States) connected to the UPLC system via an electrospray ionization (ESI) source operating in both positive and negative ionization modes. Data were acquired in MSE mode, covering a mass range of 50–1,200 Da. MS spectra were obtained using collision energies ranging from 20 to 50 eV, with a scan time of 0.5 s per spectrum. The cone voltage was set at 40 kV, the capillary voltage was 2.0 kV, and the desolvation gas flow rate was 600 L/h at 350°C. The source temperature was maintained at 120°C, with a cone gas flow rate of 50 L/h. Lock mass calibration was performed using [M + H] + (m/z 556.2771) in positive mode and [M-H]-(m/z 554.2615) in negative mode to ensure mass accuracy. Data acquisition and analysis were performed using MassLynx™ V4.1 software (Waters Corp., Milford, United States).

For metabolomic analysis, the data were preprocessed using Progenesis QI V2.1 software (Waters Corp., Milford, USA) as previously described (20). Principal component analysis (PCA) and orthogonal partial least squares-discriminant analysis (OPLS-DA) were performed using EZinfo V3.0 software (MKS Umetrics, Umea, Sweden). Variable importance in projection (VIP) values from the OPLS-DA model was used to identify potential metabolites, applying thresholds of VIP > 1, fold change (FC) > 3, and *p* < 0.05. Pathway enrichment and metabolic pathway analyses were performed using MetaboAnalyst 5.0.^1^ Heatmaps and Spearman correlation analyses were generated via the OmicShare bioinformatics platform.^2^ The correlation network of various parameters was visualized using Gephi software (version 0.9.2) as previously described (21).

### 2.9 Statistical analysis

Statistical analysis was performed using SPSS Version 29.0 (IBM Corp., Armonk, NY, United States). Data are expressed as means ± standard deviation (SD). One-way analysis of variance (ANOVA) followed by Tukey’s multiple comparison test was used to determine statistical significance. *p* < 0.05 was considered statistically significant.

## 3 Results

### 3.1 Effects of oral GGV formula on liver biochemical markers and body weight in mice

**Figure 1:**
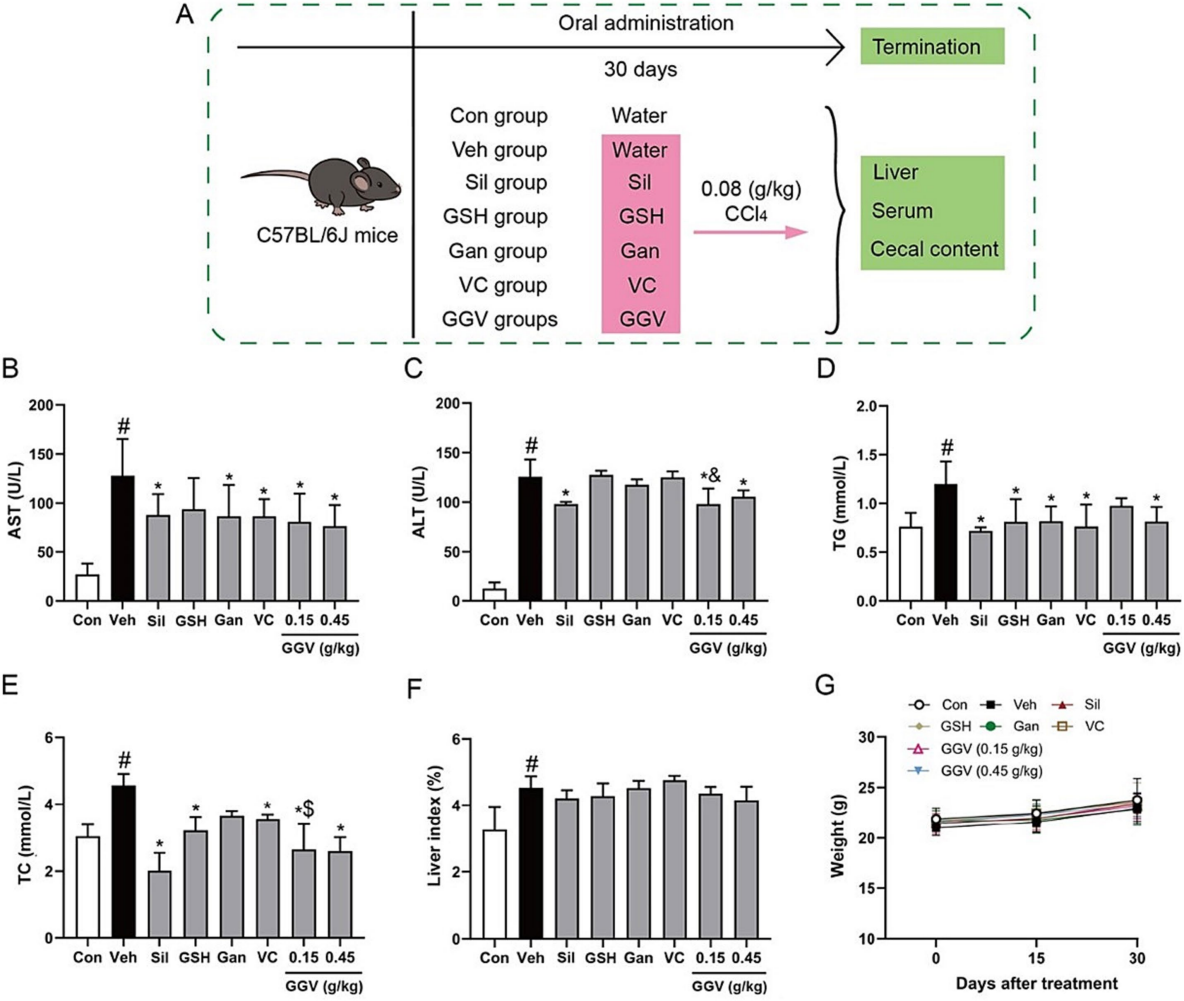
Effects of various treatments on liver function and lipid metabolism in C57BL/6J mice. **(A)** Schematic representation of the animal experimental design. The mice received oral treatments for 30 days, followed by the administration of CCl_4_ (0.08 g/kg). Silymarin served as a positive control. Serum levels of AST **(B)**, ALT **(C)**, TG **(D)**, and TC **(E)** were measured. The liver index **(F)** and body weight changes **(G)** were also assessed. Data are presented as means ± SD (*n* = 10). ^#^*p* < 0.05 compared with the control group; *p < 0.05 compared with the vehicle group; ^&^*p* < 0.05 compared with the GSH or VC group; ^$^*p* < 0.05 compared with the Gan or VC group. Veh: vehicle; Sil: silymarin; GSH: glutathione; VC: vitamin C; GGV: the formulation comprising glutathione, *Ganoderma lucidum* extract and vitamin C.

To assess the hepatoprotective effects of GGV, we designed the animal study as illustrated in Figure 1A. In the vehicle group, CCl_4_ administration significantly elevated the serum levels of AST and ALT, reflecting severe liver injury. In contrast, both the 0.15 g/kg and 0.45 g/kg GGV-treated groups showed significantly lower AST and ALT levels compared with the vehicle group (*p* < 0.05, Figures 1B,C). Notably, only GGV-treated groups, not the groups receiving individual components, demonstrated a significant reduction in ALT level. Interestingly, at the lower dose, GGV reduced ALT level more effectively than equivalent doses of glutathione or vitamin C. In addition to its hepatoprotective effects, GGV exhibited lipid-regulating properties by significantly reducing the serum TG and TC levels (*p* < 0.05), which were elevated in the vehicle group (Figures 1D,E). Compared with equivalent doses of Gan or VC, high doses of GGV significantly reduced TG level. Furthermore, GGV treatment appeared to reduce the liver index in a dose-dependent manner (Figure 1F), suggesting its potential to prevent hepatomegaly, a common consequence of liver injury. Importantly, no significant differences in body weight were observed among the groups (Figure 1G), indicating that GGV administration did not negatively impact overall body mass. These findings highlight GGV as a highly effective and safe therapeutic option for liver injury and associated metabolic disorders, offering both hepatoprotective and lipid-regulating benefits without adverse effects on body weight.

### 3.2 Pathological and antioxidant effects of GGV on the mouse liver

**Figure 2:**
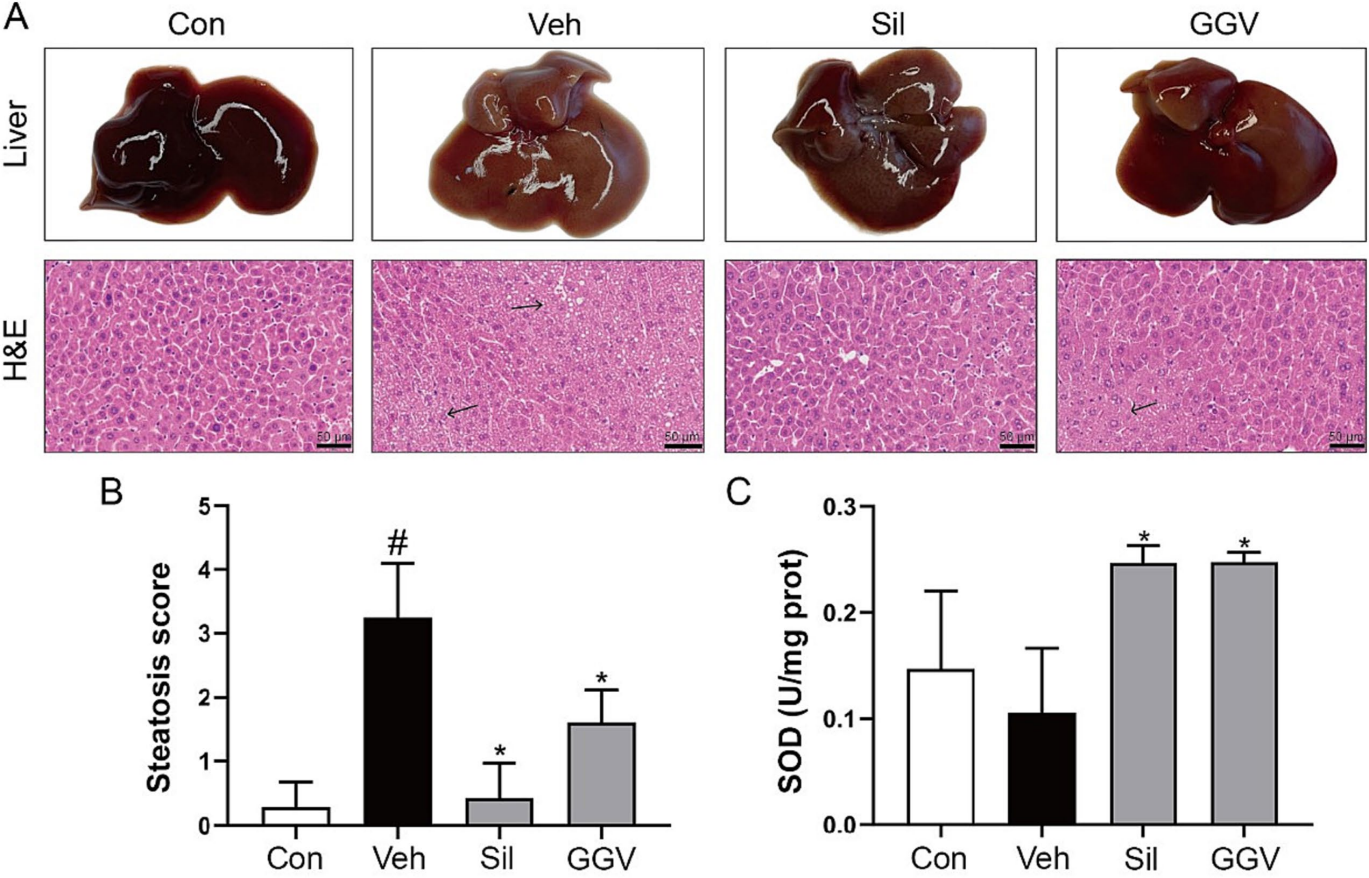
Effects of GGV on liver morphology, steatosis and antioxidant activity. **(A)** Representative images of liver morphology (top row) alongside corresponding H&E-stained liver sections (bottom row). Scale bars: 50 μm. **(B)** Quantification of the liver steatosis scores for each group. **(C)** Evaluation of SOD activity in liver tissue. Data are presented as means ± SD. ^#^*p* < 0.05 compared with the control group. *p < 0.05 compared with the vehicle group.

Figure 2A shows representative macroscopic and H&E-stained histological images of liver tissues. The vehicle group exhibited severe liver damage, including visible gross pathological changes and marked hepatocyte vacuolation (indicated by arrows), a characteristic feature of steatosis. In contrast, GGV (0.45 g/kg) treatment significantly attenuated these pathological alterations, reflected by the more normal liver appearance and reduced vacuolar degeneration in histological sections. Figure 2B shows the steatosis score, which was significantly greater in the vehicle group than in the control group (*p* < 0.05), indicating the development of fatty liver. GGV treatment significantly reduced the steatosis score (*p* < 0.05), reflecting its ability to mitigate lipid accumulation in the liver. As expected, the positive control group treated with silymarin also showed a significant reduction in steatosis. In addition to its protective effects on liver structure, GGV enhanced the liver’s antioxidant defenses. Figure 2C shows the effect of GGV on the activity of SOD, a key antioxidant enzyme. Compared with the control group, the vehicle group showed reduced SOD activity, which is indicative of oxidative stress. In contrast, both the GGV and silymarin treated groups demonstrated significantly increased SOD activity compared with the vehicle group (*p* < 0.05), suggesting that GGV enhances the antioxidant defenses of the liver. These findings demonstrate the dual role of GGV in reducing liver steatosis and improving antioxidant capacity.

### 3.3 Effects of GGV on the composition of the gut microbiota

**Figure 3:**
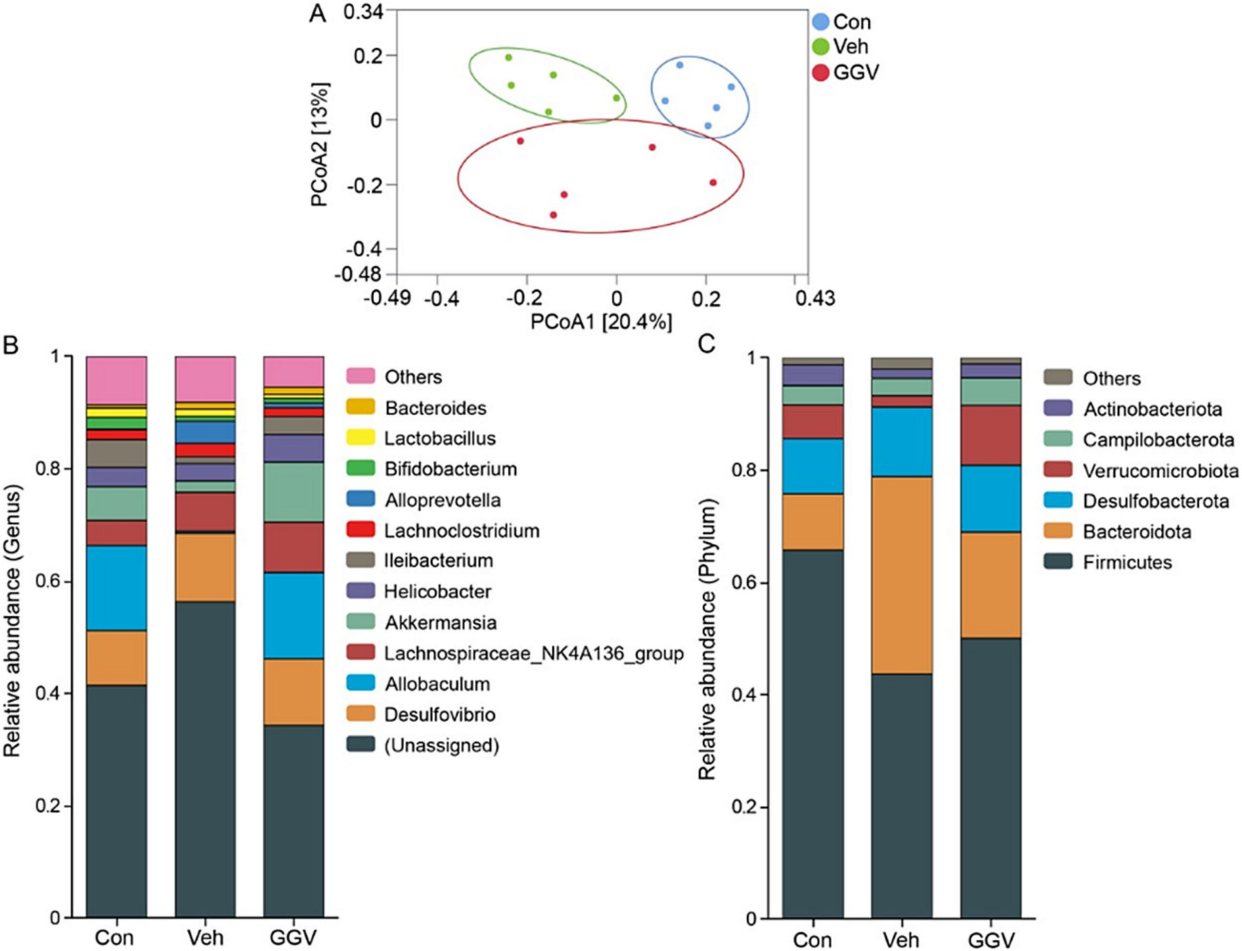
Modulation of the gut microbiota following GGV treatment. **(A)** PCoA plot based on Bray-Curtis distances showing the separation of microbial communities among groups. Bar charts depict the relative abundance of bacterial genera **(B)** and phyla **(C)** across the different groups.

The gut microbiota plays a crucial role in maintaining liver health, and its modulation represents a promising therapeutic strategy for managing liver injury (22). To evaluate the effects of GGV (0.45 g/kg) treatment on gut microbiota, we analyzed fecal samples by sequencing the V3 − V4 region of the 16S rRNA gene. PCoA plot (Figure 3A) revealed distinct clustering of gut microbiota composition among the groups, with clear separations observed between the control, vehicle, and GGV-treated groups. These results suggest that while CCl_4_ treatment disrupted gut microbial composition, GGV treatment restored it closer to the baseline of the control group. Further analysis of gut microbiota composition at both the phylum and genus levels (Figures 3B,C) revealed substantial changes. At the genus level (Figure 3B), CCl_4_ administration decreased the abundance of beneficial genera, including *Allobaculum*, *Akkermansia*, and *Ileibacterium*, while increasing the abundance of *Alloprevotella*. At the phylum level, *Firmicutes* and *Bacteroidota* were the dominant phyla in all the groups, accounting for more than 60% of the microbial community. Notably, compared with the vehicle group, GGV group exhibited an increased *Firmicutes*/*Bacteroidota* ratio (Figure 3C). This ratio showed a negative correlation with liver enzymes such as ALT and AST, indicating its potential involvement in regulating liver health (23).

### 3.4 Correlations between the gut microbiota and liver biochemical parameters

**Figure 4:**
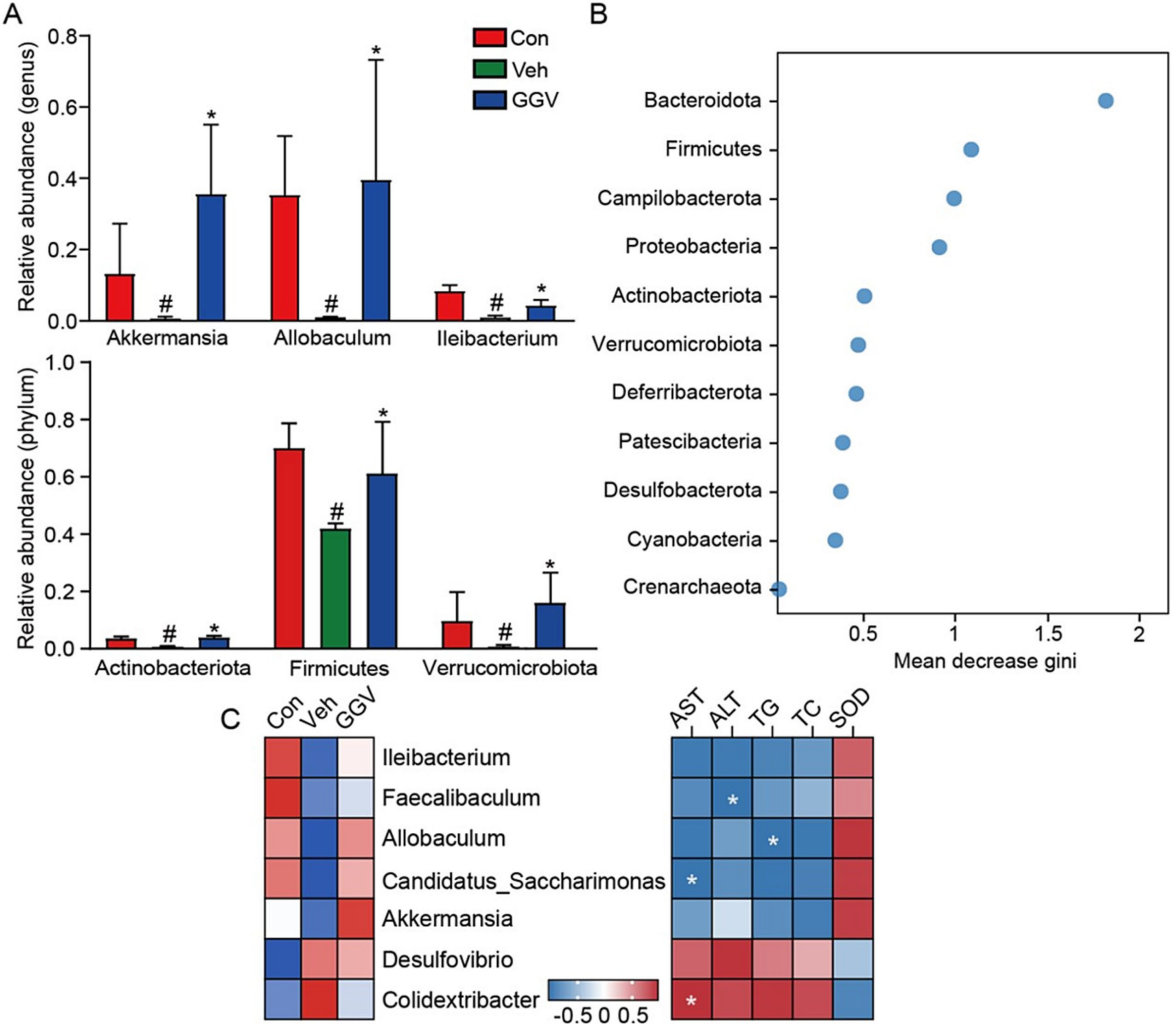
Effects of GGV on the gut microbial composition in CCl_4_-induced liver injury mouse model. **(A)** Relative abundance of the microbial taxa at the genus (top) and phylum (bottom) levels. ^#^*p* < 0.05 compared with the control group. *p < 0.05 compared with the vehicle group. **(B)** The Random Forest analysis identified key phyla markers distinguishing the experimental groups. **(C)** Heatmap illustrating the relative abundance of key bacterial genera (left panel) and their correlations with metabolic parameters (right panel), including AST, ALT, TG, TC, and SOD. Positive correlations are indicated in red, while negative correlations are shown in blue. Statistically significant correlations (*p* < 0.05) are marked with white asterisks (*) within the squares.

Compared with the control group, the vehicle group exhibited a significant reduction in gut microbiota diversity at both the genus and phylum levels. Notably, treatment with GGV formula (0.45 g/kg) effectively reversed these changes, restoring microbial balance (Figure 4A). For instance, GGV treatment significantly increased the abundance of *Akkermansia*, a beneficial microorganism known for its critical role in promoting health (24). Further analysis using the Random Forest model identified key phyla contributing to the observed differences among the experimental groups, as evidenced by their high mean decrease in Gini scores (Figure 4B). Correlation analysis revealed notable relationships between specific microbial genera and metabolic health markers, including AST, ALT, TG, TC, and SOD (Figure 4C). Specifically, significant negative correlations were observed: *Faecalibaculum*, *Alloprevotella*, and *Candidatus Saccharimonas* were significantly negatively correlated with ALT, TG, and AST, respectively. In contrast, *Colidextribacter* was significantly positively correlated with AST. These associations suggest that these microbial genera may play distinct roles in regulating hepatic health and metabolic function.

### 3.5 Metabolite identification and multivariate statistical analysis

**Figure 5:**
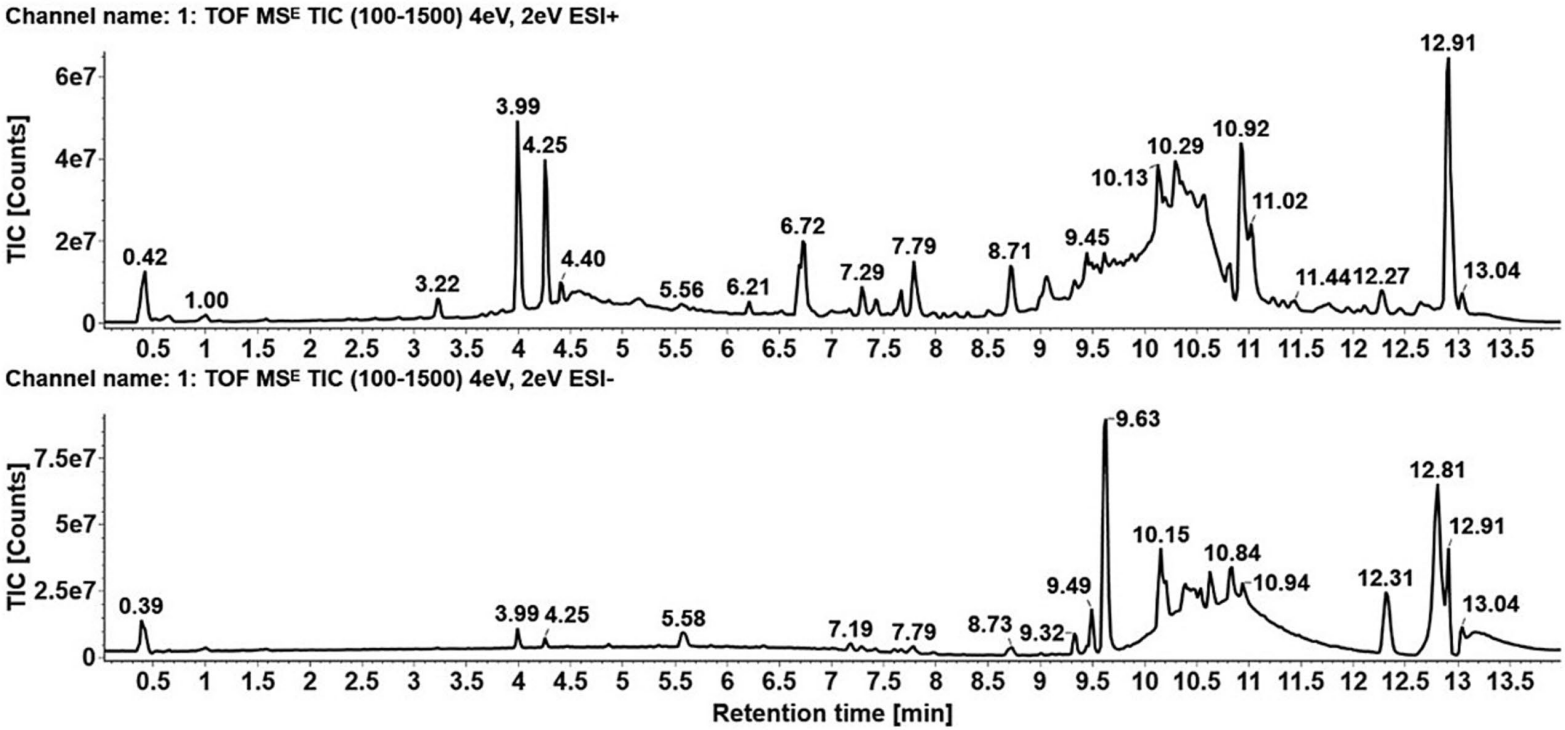
Chromatographic analysis of GGV components and metabolites via UPLC-Q-TOF-MS. Chromatograms display retention times (x-axis) for each peak and ion counts (y-axis), which represent the relative abundance of each component or metabolite. The upper chromatogram shows results from the positive ion mode, while the lower chromatogram displays results from the negative ion mode.

We utilized UPLC-Q/TOF-MS/MS (Agilent Technologies, Santa Clara, CA, United States) to analyze blood metabolites in both positive and negative ionization modes. As illustrated in Figure 5, the positive ion mode revealed several prominent peaks corresponding to metabolites eluted at specific retention times, including 3.99, 4.25, 10.92, and 12.91 min. The most substantial peak, observed at 12.91 min, suggested a major metabolite with high abundance. In contrast, the negative ion mode presented a distinct chromatographic profile with primary peaks identified at 9.63, 10.15, and 12.81 min. The details of the identified metabolites are provided in Supplementary Table S1. We detected Ganolucidic acid A in the serum of metaplastic study. This bioactive triterpene compound is derived from *Ganoderma lucidum*. To ensure analytical precision, quality control (QC) samples were used to monitor and validate the consistency of the analysis.

**Figure 6:**
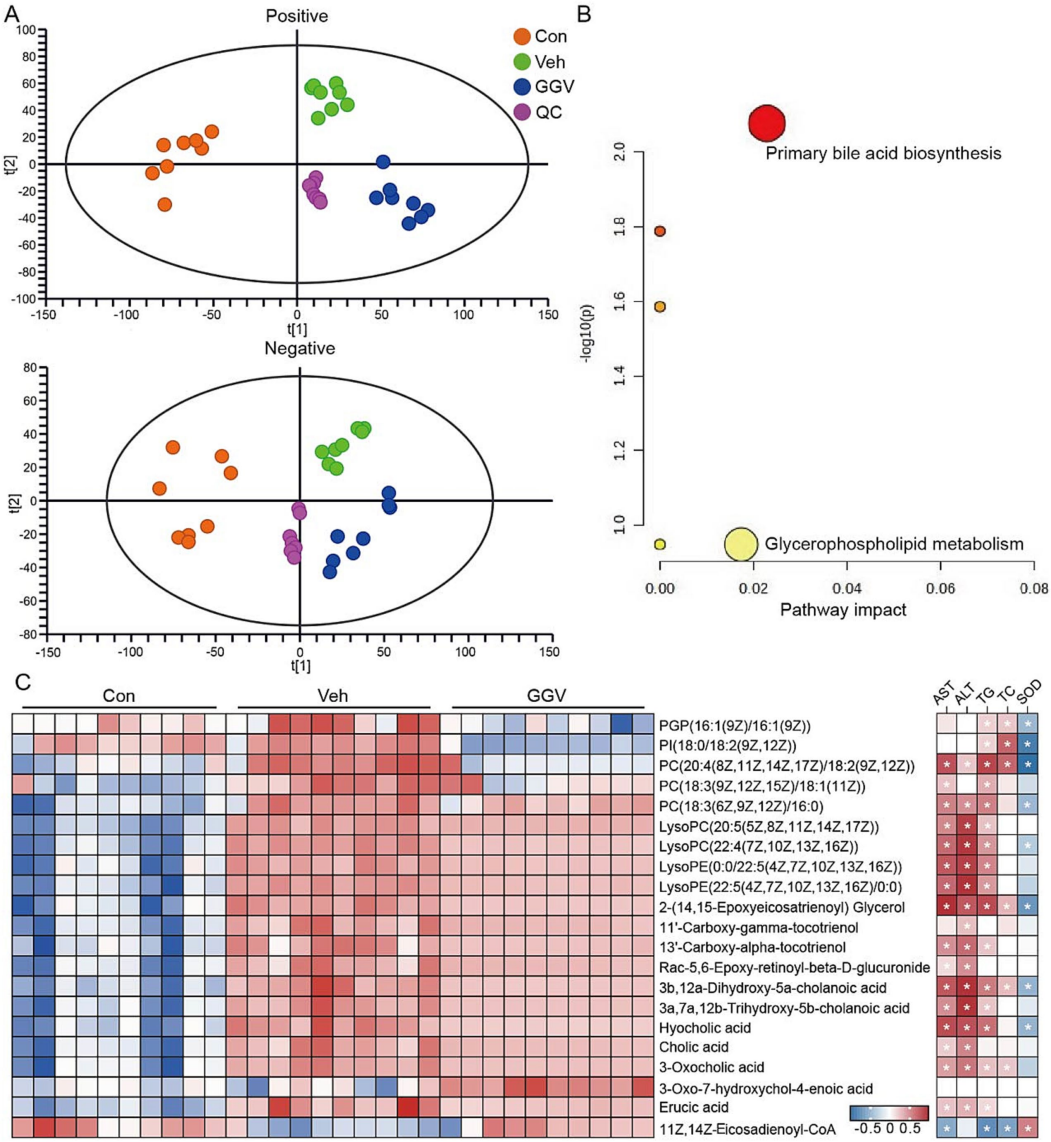
Metabolic profile and correlations with hepatic biochemical parameters following treatment. **(A)** PCA score plots illustrating the metabolic differences among the control (orange), vehicle (green), and GGV (blue) groups in both negative and positive ionization modes. **(B)** Pathway analysis revealed statistically significant pathways, with color intensity indicating *p* values. **(C)** Heatmap showing z score-normalized metabolite intensities across the groups and their correlations with biochemical parameters (AST, ALT, TG, TC, and SOD). The blue squares indicate negative correlations, while the red squares represent positive correlations. White asterisks (*) within the squares indicate statistically significant correlations (*p* < 0.05).

QC samples displayed tight clustering in both ionization modes (Figure 6A), indicating the robustness and reproducibility of the measurement protocol with minimal variability. PCA of the metabolic profiles revealed distinct clustering among the control, vehicle, and GGV-treated groups, indicating metabolic shifts. A total of 21 metabolites were significantly altered in the GGV-treated group (*p* < 0.05, fold change > 3, VIP > 1), indicating a substantial metabolic response to GGV treatment. Pathway enrichment analysis (Figure 6B) revealed notable perturbations in primary bile acid biosynthesis and glycerophospholipid metabolism, with thresholds of −log10(P) > 1 and pathway impact values exceeding 0.01. These pathways were the most affected, suggesting that GGV treatment induces targeted metabolic reprogramming, particularly in lipid and bile acid metabolism.

Figure 6C provides a detailed heatmap visualization of the identified metabolites, showing a marked increase in the levels of phospholipids (PGP, PI, PCs, LysoPCs, and LysoPEs) in the vehicle group. These phospholipids were positively correlated with the hepatic biomarkers AST and TG, suggesting a link between elevated lipid metabolites and impaired liver function. Conversely, GGV treatment significantly attenuated the levels of key lipid metabolites, including LysoPCs, LysoPE, 2-(14,15-epoxyeicosatrienoyl) glycerol, and various bile acids, such as 3β, 12α-dihydroxy-5α-cholanoic acid, 3α, 7α, 12β-trihydroxy-5β-cholanoic acid, hyocholic acid, cholic acid, and 3-oxocholic acid. These reductions aligned with the decreased levels of AST, ALT, TG, and TC in the GGV-treated group. Notably, the fatty acyl-CoA species 11Z, 14Z-eicosadienoyl-CoA, which was depleted in the vehicle group, exhibited partial recovery in the GGV group, which was correlated with improvements in hepatic markers (AST, TG, TC, and SOD). Collectively, these findings indicate that GGV treatment modulates the serum metabolite profile, particularly lipid and bile acid metabolism, supporting a hepatoprotective role for GGV.

### 3.6 Correlations between metabolites and the gut microbiota

**Figure 7:**
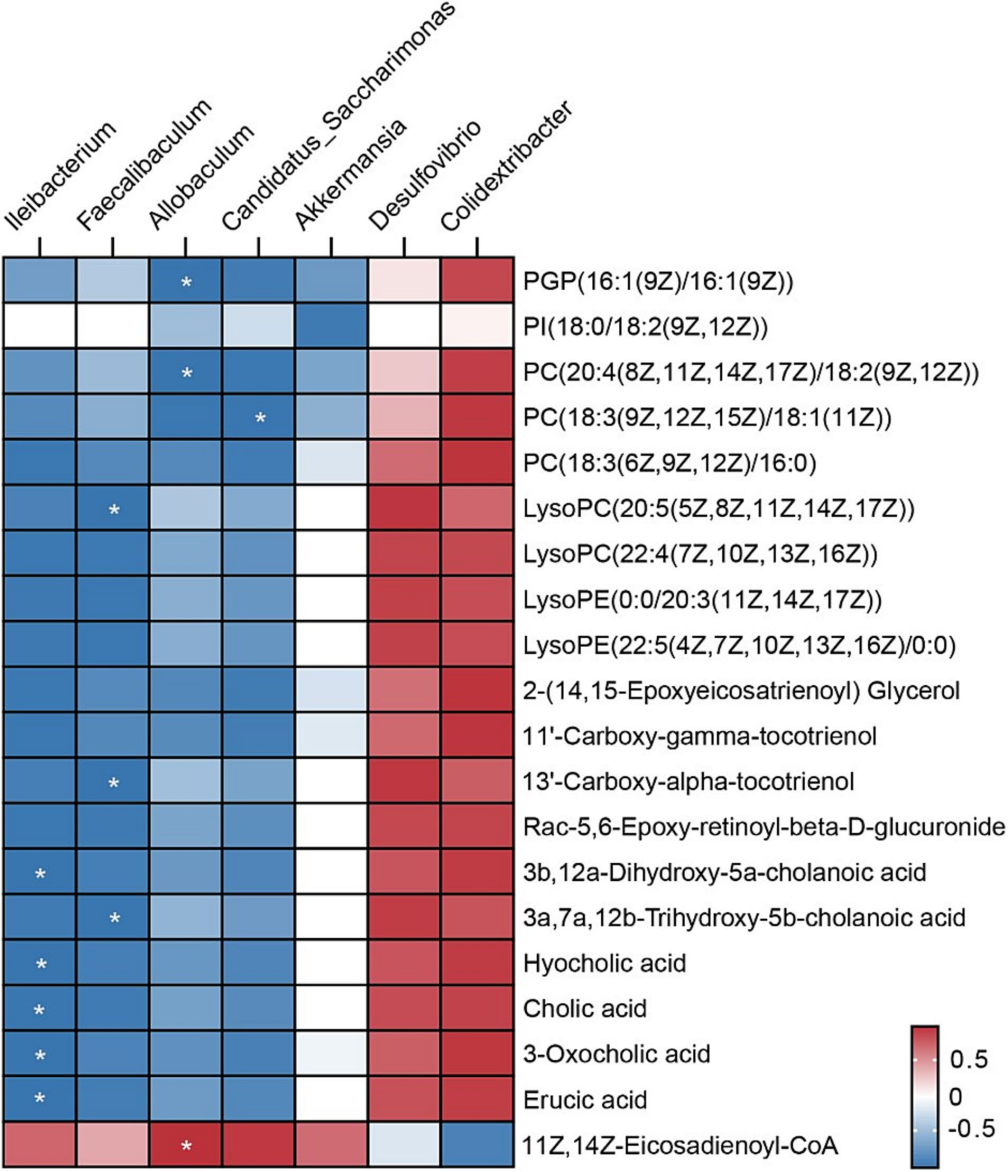
Correlation heatmap of the gut microbiota and metabolites. The heatmap illustrates Spearman’s rank correlation coefficients, with color gradients indicating the strength of the correlations. Red squares represent positive correlations, while blue squares indicate negative correlations. The intensity of the color reflects the magnitude of the correlation. White asterisks (*) within the squares indicate statistically significant correlations (*p* < 0.05).

To explore the relationships between gut microbiota composition and serum metabolites, Spearman’s rank correlation coefficient was calculated. As illustrated in Figure 7, a correlation analysis revealed associations between 20 differentially abundant metabolites and 7 distinct microbial genera, which formed distinct clusters. Among the seven genera analyzed, four microorganisms exhibited significant positive or negative correlations (*p* < 0.05) with at least one metabolite, including phosphatidylglycerol phosphate (PGP), phosphatidylcholine (PC), and lysophosphatidylcholine (LysoPC). For instance, *Allobaculum* displayed negative correlations with metabolites such as PGP and PC, but was positively correlated with the fatty acid CoA derivative 11Z, 14Z-Eicosadienoyl-CoA. These findings suggest that *Allobaculum* may play diverse metabolic roles and contribute to varying metabolite profiles under conditions of CCl_4_-induced hepatic injury in mice. Notably, most of the serum metabolites were negatively correlated with *Ileibacterium*, *Faecalibaculum*, *Allobaculum*, *Candidatus Saccharimonas*, and *Akkermansia*, while positive correlations were predominantly observed with *Desulfovibrio* and *Colidextribacter*.

## 4 Discussion

**Figure 8:**
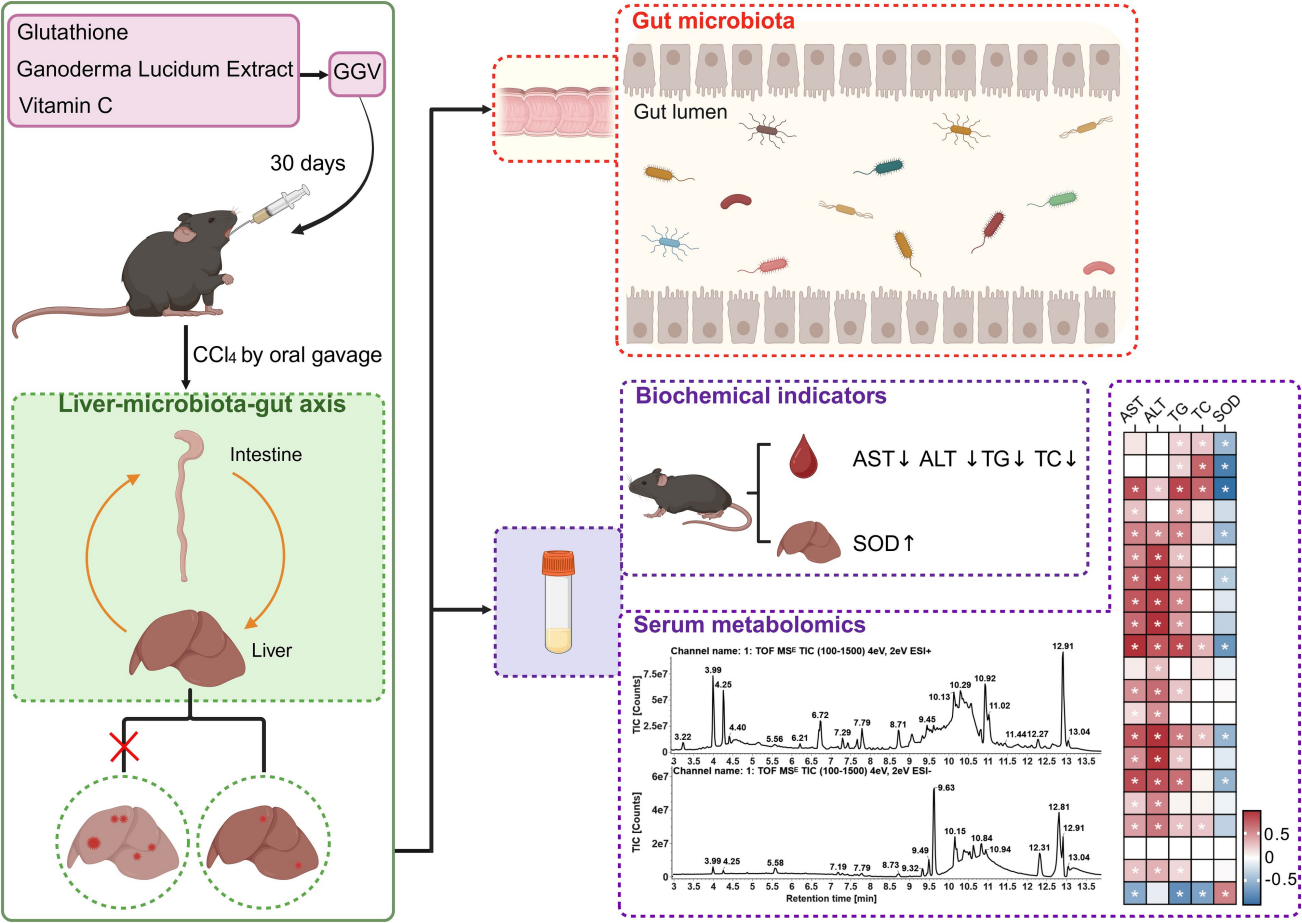
Schematic overview of the pharmacological actions of GGV in alleviating CCl_4_-induced acute liver injury in mice through modulation of the liver-microbiota-gut axis. GGV administration for 30 days improved the levels of serum and liver biochemical markers, including reductions in AST, ALT, TG, and TC levels, along with an increase in SOD activity (top right), indicating enhanced liver function and reduced oxidative stress. Additionally, GGV treatment enhanced gut microbiota diversity (middle right), suggesting a beneficial shift in microbial composition that supports liver health. Key serum metabolites were identified using UPLC-Q/TOF-MS/MS, with a correlation heatmap (bottom right) illustrating the relationships between these metabolites and critical biochemical markers, and highlighting the interconnected metabolic pathways influenced by GGV treatment in mitigating liver injury.

This study demonstrated that the GGV formula exhibits hepatoprotective effects in a CCl_4_-induced liver injury model in mice. Our results revealed that the GGV formula and its individual components significantly reduced the serum AST, TG, and TC levels. Additionally, GGV treatment also reduced liver steatosis and enhanced SOD activity, reflecting improved antioxidant defense. Mechanistic investigations indicated that these protective effects of GGV are, at least in part, mediated by the alterations of gut microbiota composition and the promotion of beneficial metabolite production (Figure 8). Taken together, these findings support the potential of GGV as functional food for liver protection, possibly through the modulation of the liver-microbiota-gut axis.

In terms of the component combination, we observed interesting protective effects on liver function. Our results demonstrated that the GGV formula, along with its individual components, significantly reduced serum levels of AST, TG, and TC. Notably, only the complete GGV formula effectively reduced ALT levels, a critical biomarker of liver damage. Furthermore, at lower doses, the GGV formula was more effective in reducing ALT levels compared to equivalent doses of glutathione or VC. In addition, at higher doses, the GGV formula significantly reduced TG levels compared to the equivalent doses of glutathione or VC. These findings suggest potential synergistic effects, which are consistent with previous studies highlighting the antioxidant synergy between vitamin C and glutathione (25). To explore the active compositions of *Ganoderma lucidum* extract, its ingredients were identified in the blood samples of mice in the intervention group. Among these, it has been previously reported that *Ganoderma lucidum* contains polysaccharides, alkaloids, and triterpenoids, which have demonstrated therapeutic effects in the treatment of liver diseases (26). Specifically, *Ganoderma lucidum* polysaccharides have been reported to inhibit liver fibrosis (27). In addition, ganoderic acids, another bioactive components in *Ganoderma lucidum*, have exhibited protective effects against alcohol-induced liver injury (28).

Currently, the pathogenic mechanisms underlying CCl_4_-induced hepatic injury are not been fully elucidated, but they are likely to be multi-factorial. CCl_4_ is a well-known hepatotoxin that induces oxidative stress, leading to liver injury and fibrosis (29). It is logical to speculate that antioxidants could confer hepatoprotection. Non-enzymatic antioxidants, such as vitamin C and glutathione, play crucial roles in neutralizing reactive oxygen species (ROS) and protecting cells from oxidative stress. Vitamin C directly scavenges free radicals, while glutathione serves a dual role, acting as a direct antioxidant and regenerating other antioxidants, thereby bolstering the overall defense against oxidative damage (25, 30, 31). Lipophilic antioxidants, such as vitamin E and carotenoids, protect cellular membranes by preventing lipid peroxidation, thus safeguarding cellular structures from oxidative stress. The liver also relies on its enzymatic antioxidant system, which includes superoxide dismutase (SOD), catalase (CAT), and glutathione peroxidase (GPx). These enzymes work together to neutralize ROS. SOD converts superoxide radicals into hydrogen peroxide, which is then detoxified by CAT and GPx, ensuring effective cellular protection against oxidative damage (32, 33). Additionally, phenolic compounds have been shown to directly scavenge ROS, reducing oxidative stress. By acting as electron donors, these compounds can reduce Fe^3+^ to Fe^2+^, enhancing the cell redox buffering capacity. They also support the antioxidant system by enhancing the activity of enzymes such as APX and GR, which mitigate H₂O₂-induced membrane damage. Through enzyme regulation, phenolic compounds inhibit PPO/POD activity while activating APX/GR, maintaining redox balance (34). In the present study, we found that GGV treatment increased SOD activity and increased the abundances of *Allobaculum* and *Akkermansia*, both of which are positively correlated with SOD levels, indicating enhanced antioxidant capacity. These findings underscore the importance of antioxidants in liver health and support the role of the GGV formula in enhancing antioxidative responses for liver protection.

Besides the antioxidant-based strategies for live protection, modulation of the gut microbiota has emerged as a promising strategy for alleviating symptoms of CCl_4_-induced acute liver injury (35). A systematic review highlighted the potential of modifying intestinal microbiota as a potential therapeutic strategy for liver disease (36). In this study, we found that GGV administration effectively reversed microbial dysbiosis induced by CCl_4_ exposure. This effect was characterized by the enrichment of beneficial bacterial taxa, including *Akkermansia*, *Allobaculum*, and *Firmicutes*. Among these, *Akkermansia* is widely recognized for its hepatoprotective effects, including reducing liver fibrosis and inflammation in alcohol-induced liver injury model (37). It improves biochemical marker levels, reduces inflammatory cytokine levels, and ameliorates histopathological damage (38). Furthermore, *Akkermansia* has been implicated in alleviating metabolic dysfunction associated with fatty liver disease by enhancing L-asparagine metabolism via the gut-liver axis (39). Similarly, *Allobaculum* is associated with reversing hepatic lesions, reducing fat accumulation, and alleviating oxidative stress in liver tissues (40). Another study demonstrated that a traditional herbal formula enriched with *Allobaculum* improved gut microbial composition and liver function in a model of nonalcoholic fatty liver disease (41). *Firmicutes*, on the other hand, contribute to gut barrier integrity and regulate metabolic pathways, with increased abundance linked to improved liver health and reduced fibrosis and inflammation (42, 43). Based on our findings and the existing literature, the hepatoprotective effects of GGV may, at least in part, be attributed to restoring microbial balance and enriching beneficial bacterial taxa.

Gut microbiota also plays a critical role in maintaining host health, particularly by regulating metabolism (44). Extensive evidence has revealed that GGV influences bile acid metabolism, a key factor associated with microbial metabolic stability (45). *Alistipes*, identified as a potential producer of short-chain fatty acids (SCFA), contribute to modulating inflammatory responses, with decreased levels linked to hepatic fibrosis (46). SCFA production by certain gut microbes further supports liver protection through their anti-inflammatory effects and maintenance of mucosal integrity (47). The increased SCFA production regulated by GGV administration likely aids in reducing chronic inflammation, thereby reinforcing the hepatoprotective properties of GGV. In addition, bile acids shape the composition of the gut microbiota and are transformed by gut bacteria, creating a feedback mechanism essential for liver and gut health (48). Primary bile acids are reabsorbed and recirculated through the enterohepatic system, while gut microbiota converts them into secondary bile acids, which regulate host metabolism and immune responses (49). Modulation of this bile acid cycle by GGV suggests its therapeutic potential in stabilizing the liver-microbiota-gut axis and enhancing liver detoxification processes. These findings highlight the need for future research on dietary components that influence bile acid metabolism and gut microbiota composition, which may reveal new strategies for treating liver diseases (50).

The therapeutic potential of GGV is further underscored by its role in modulating the liver-microbiota-gut axis, a complex system of interactions in which gut-derived metabolites influence liver function, while the liver, in turn, regulates the gut microbiota through bile acid release. This reciprocal relationship is critical, as disruptions in the gut microbiota are associated with liver diseases (51). Modulation of bile acid metabolism by GGV may stabilize microbial communities and improve metabolic homeostasis, particularly by enhancing lipid regulation. Furthermore, interventions targeting the liver-microbiota-gut axis may offer novel approach for treating specific toxicities, such as arsenic exposure in poultry by promoting gut microbiota balance to improve liver function and reduce toxicity (52). Overall, GGV shows promise in managing liver injuries and related metabolic dysfunctions by reinforcing the bidirectional communication within the liver-microbiota-gut axis. Despite these promising findings supporting the therapeutic potential of GGV, several limitations of this study must be acknowledged. First, the mechanisms underlying interactions between serum metabolites and the gut microbiota are complex and not yet fully understood. Further experimental validation is needed to gain a deeper understanding of these relationships. Second, although the current study provides a chemical characterization of the phytotherapeutic agents used, it does not determine whether a specific biomarker of *Ganoderma lucidum* is present in liver tissue. This aspect should be further clarified in future investigations. Finally, the specific antioxidant mechanisms involved need further exploration. For example, measuring multiple antioxidant markers across different experimental groups could provide valuable insight into the potential synergistic effects of the active ingredients within GGV.

## 5 Conclusion

Using a CCl_4_-induced acute liver injury model, this study demonstrated that GGV provides protective effects, as evidenced by improvements in ALT and AST levels and histopathological outcomes in mice. According to the criteria in the Technical Standards for the Testing and Assessment of Health Foods in China, GGV exhibits hepatoprotective properties against chemically induced liver damage. The findings of this study suggest that GGV may alleviate CCl_4_-induced acute liver injury, potentially through modulation of the liver-microbiota-gut axis. These results underscore the potential of GGV for developing innovative therapeutic strategies for acute liver injury.

## Data Availability

The raw data supporting the conclusions of this article will be made available by the authors, without undue reservation.

## Footnotes

^1^ http://metpa.metabolomics.ca

^2^ http://www.omicshare.com/tools

## References

[1] Devarbhavi H Asrani SK Arab JP Nartey YA Pose E Kamath PS. Global burden of liver disease: 2023 update. J Hepatol. (2023) 79:516–37. doi: 10.1016/j.jhep.2023.03.017, PMID: 36990226

[2] Karlsen TH Sheron N Zelber-Sagi S Carrieri P Dusheiko G Bugianesi E. The EASL-lancet liver commission: protecting the next generation of Europeans against liver disease complications and premature mortality. Lancet. (2022) 399:61–116. doi: 10.1016/s0140-6736(21)01701-3, PMID: 34863359

[3] Dai C Xiao X Li D Tun S Wang Y Velkov T. Chloroquine ameliorates carbon tetrachloride-induced acute liver injury in mice via the concomitant inhibition of inflammation and induction of apoptosis. Cell Death Dis. (2018) 9:1164. doi: 10.1038/s41419-018-1136-2, PMID: 30478280 PMC6255886

[4] Li YJ Liu RP Ding MN Zheng Q Wu JZ Xue XY. Tetramethylpyrazine prevents liver fibrotic injury in mice by targeting hepatocyte-derived and mitochondrial DNA-enriched extracellular vesicles. Acta Pharmacol Sin. (2022) 43:2026–41. doi: 10.1038/s41401-021-00843-w, PMID: 35027662 PMC9343419

[5] Bilzer M Baron A Schauer R Steib C Ebensberger S Gerbes AL. Glutathione treatment protects the rat liver against injury after warm ischemia and Kupffer cell activation. Digestion. (2002) 66:49–57. doi: 10.1159/000064415, PMID: 12379815

[6] El Sherif F Khattab S Ibrahim AK Ahmed SA. Improved Silymarin content in elicited multiple shoot cultures of *Silybum Marianum* L. Physiol Mol Biol Plants. (2013) 19:127–36. doi: 10.1007/s12298-012-0141-7, PMID: 24381444 PMC3550681

[7] Kadoglou NPE Panayiotou C Vardas M Balaskas N Kostomitsopoulos NG Tsaroucha AK. A comprehensive review of the cardiovascular protective properties of Silibinin/Silymarin: a new kid on the block. Pharmaceuticals (Basel). (2022) 15:538. doi: 10.3390/ph15050538, PMID: 35631363 PMC9145573

[8] Xie Y Zhang D Zhang J Yuan J. Metabolism, transport and drug-drug interactions of Silymarin. Molecules. (2019) 24:3693. doi: 10.3390/molecules24203693, PMID: 31615114 PMC6832356

[9] Wu J-W Lin L-C Tsai T-H. Drug-drug interactions of Silymarin on the perspective of pharmacokinetics. J Ethnopharmacol. (2009) 121:185–93. doi: 10.1016/j.jep.2008.10.036, PMID: 19041708

[10] Parés A Planas R Torres M Caballería J Viver JM Acero D. Effects of Silymarin in alcoholic patients with cirrhosis of the liver: results of a controlled, double-blind, randomized and multicenter trial. J Hepatol. (1998) 28:615–21. doi: 10.1016/s0168-8278(98)80285-7 9566830

[11] Wei F Liu SK Liu XY Li ZJ Li B Zhou YL. Meta-analysis: Silymarin and its combination therapy for the treatment of chronic hepatitis B. Eur J Clin Microbiol Infect Dis. (2013) 32:657–69. doi: 10.1007/s10096-012-1789-1, PMID: 23247631

[12] Di Paola R Modafferi S Siracusa R Cordaro M D'Amico R Ontario ML. S-acetyl-glutathione attenuates carbon tetrachloride-induced liver injury by modulating oxidative imbalance and inflammation. Int J Mol Sci. (2022) 23:4429. doi: 10.3390/ijms23084429, PMID: 35457246 PMC9024626

[13] Su M Chen H Wei C Chen N Wu W. Potential protection of vitamin C against liver-lesioned mice. Int Immunopharmacol. (2014) 22:492–7. doi: 10.1016/j.intimp.2014.07.034, PMID: 25116224

[14] Chen TQ Wu JG Kan YJ Yang C Wu YB Wu JZ. Antioxidant and Hepatoprotective activities of crude polysaccharide extracts from Lingzhi or Reishi medicinal mushroom, *Ganoderma lucidum* (Agaricomycetes), by ultrasonic-circulating extraction. Int J Med Mushrooms. (2018) 20:581–93. doi: 10.1615/IntJMedMushrooms.2018026536, PMID: 29953354

[15] Ren A Wu T Wang Y Fan Q Yang Z Zhang S. Integrating animal experiments, mass spectrometry and network-based approach to reveal the sleep-improving effects of Ziziphi Spinosae semen and γ-aminobutyric acid mixture. Chin Med. (2023) 18:99. doi: 10.1186/s13020-023-00814-9, PMID: 37573423 PMC10422734

[16] Chen R Lian Y Wen S Li Q Sun L Lai X. Shibi tea (*Adinandra nitida*) and Camellianin a alleviate ccl(4)-induced liver injury in C57bl-6j mice by attenuation of oxidative stress, inflammation, and apoptosis. Nutrients. (2022) 14:3037. doi: 10.3390/nu14153037, PMID: 35893891 PMC9332116

[17] Wei J Wang S Huang J Zhou X Qian Z Wu T. Network medicine-based analysis of the Hepatoprotective effects of *Amomum villosum* Lour. On alcoholic liver disease in rats. Food Sci Nutr. (2024) 12:3759–73. doi: 10.1002/fsn3.4046, PMID: 38726425 PMC11077240

[18] Huang T Jiang J Cao Y Huang J Zhang F Cui G. Camellia oil (*Camellia Oleifera* Abel.) treatment improves high-fat diet-induced atherosclerosis in apolipoprotein E (Apoe)^−/−^ mice. Biosci Microbiota Food Health. (2023) 42:56–64. doi: 10.12938/bmfh.2022-005, PMID: 36660600 PMC9816045

[19] Wu Z Luo W Kuang S Zhao L Wang Y He Y. Integrated gut microbiota and serum metabolomic analysis to investigate the mechanism of the immune-enhancing effect of SVS formula in mice. J Funct Foods. (2024) 122:106468. doi: 10.1016/j.jff.2024.106468

[20] Wang Y Zou Z Wang S Ren A Ding Z Li Y. Golden bile powder prevents drunkenness and alcohol-induced liver injury in mice via the gut microbiota and metabolic modulation. Chin Med. (2024) 19:39. doi: 10.1186/s13020-024-00912-2, PMID: 38431607 PMC10908100

[21] Han H Zhao C Liu M Zhu H Meng F Zhang Y. Mitochondrial complex I inhibition by Homoharringtonine: a novel strategy for suppression of chronic myeloid leukemia. Biochem Pharmacol. (2023) 218:115875. doi: 10.1016/j.bcp.2023.115875, PMID: 37871881

[22] Juanola O Hassan M Kumar P Yilmaz B Keller I Simillion C. Intestinal microbiota drives cholestasis-induced specific hepatic gene expression patterns. Gut Microbes. (2021) 13:1–20. doi: 10.1080/19490976.2021.1911534, PMID: 33847205 PMC8049203

[23] Hassan N El-Masry S Nageeb A Hussieny ME Khalil A Aly M. Correlation between gut microbiota, its metabolic products, and their association with liver enzymes among sample of Egyptian females. Open Access Maced J Med Sci. (2021) 10:1797–804. doi: 10.3889/oamjms.2022.7909

[24] Cani PD Depommier C Derrien M Everard A de Vos WM. *Akkermansia Muciniphila*: paradigm for next-generation beneficial microorganisms. Nat Rev Gastroenterol Hepatol. (2022) 19:625–37. Epub 2022/06/01. doi: 10.1038/s41575-022-00631-9, PMID: 35641786

[25] Shalan MG. Mitigating Lead acetate-induced histopathologic and physiologic disorders in rats receiving vitamin C and glutathione supplement. Heliyon. (2025) 11:e41256. doi: 10.1016/j.heliyon.2024.e41256, PMID: 39801977 PMC11719362

[26] Ahmad R Riaz M Khan A Aljamea A Algheryafi M Sewaket D. *Ganoderma lucidum* (Reishi) an edible mushroom; a comprehensive and critical review of its nutritional, cosmeceutical, mycochemical, pharmacological, clinical, and toxicological properties. Phytother Res. (2021) 35:6030–62. Epub 2021/08/20. doi: 10.1002/ptr.7215, PMID: 34411377

[27] Chen C Chen J Wang Y Fang L Guo C Sang T. *Ganoderma lucidum* polysaccharide inhibits HSC activation and liver fibrosis via targeting inflammation, apoptosis, cell cycle, and ECM-receptor interaction mediated by TGF-β/Smad signaling. Phytomedicine. (2023) 110:154626. doi: 10.1016/j.phymed.2022.154626, PMID: 36603342

[28] Cao YJ Huang ZR You SZ Guo WL Zhang F Liu B. The protective effects of Ganoderic acids from *Ganoderma lucidum* fruiting body on alcoholic liver injury and intestinal microflora disturbance in mice with excessive alcohol intake. Food Secur. (2022) 11:949. doi: 10.3390/foods11070949, PMID: 35407036 PMC8997615

[29] Unsal V Cicek M Sabancilar İ. Toxicity of carbon tetrachloride, free radicals and role of antioxidants. Rev Environ Health. (2021) 36:279–95. doi: 10.1515/reveh-2020-0048, PMID: 32970608

[30] Averill-Bates DA. The antioxidant glutathione. Vitam Horm. (2023) 121:109–41. doi: 10.1016/bs.vh.2022.09.002 36707132

[31] Vašková J Kočan L Vaško L Perjési P. Glutathione-related enzymes and proteins: a review. Molecules. (2023) 28:1447. doi: 10.3390/molecules28031447, PMID: 36771108 PMC9919958

[32] Mooli RGR Mukhi D Ramakrishnan SK. Oxidative stress and redox signaling in the pathophysiology of liver diseases. Compr Physiol. (2022) 12:3167–92. doi: 10.1002/cphy.c200021, PMID: 35578969 PMC10074426

[33] Allameh A Niayesh-Mehr R Aliarab A Sebastiani G Pantopoulos K. Oxidative stress in liver pathophysiology and disease. Antioxidants (Basel). (2023) 12:1653. doi: 10.3390/antiox12091653, PMID: 37759956 PMC10525124

[34] Zha Z Tang R Wang C Li Y-l Liu S Wang L. Riboflavin inhibits browning of fresh-cut apples by repressing phenolic metabolism and enhancing antioxidant system. Postharvest Biol Technol. (2022) 187:111867. doi: 10.1016/j.postharvbio.2022.111867

[35] Xu X Liu S Zhao Y Wang M Hu L Li W. Combination of *Houttuynia Cordata* polysaccharide and *Lactiplantibacillus plantarum* P101 alleviates acute liver injury by regulating gut microbiota in mice. J Sci Food Agric. (2022) 102:6848–57. doi: 10.1002/jsfa.12046, PMID: 35639719

[36] Hsu CL Schnabl B. The gut-liver Axis and gut microbiota in health and liver disease. Nat Rev Microbiol. (2023) 21:719–33. doi: 10.1038/s41579-023-00904-3, PMID: 37316582 PMC10794111

[37] Grander C Adolph TE Wieser V Lowe P Wrzosek L Gyongyosi B. Recovery of ethanol-induced *Akkermansia Muciniphila* depletion ameliorates alcoholic liver disease. Gut. (2018) 67:891–901. doi: 10.1136/gutjnl-2016-313432, PMID: 28550049

[38] Keshavarz Aziziraftar S Bahrami R Hashemi D Shahryari A Ramezani A Ashrafian F. The beneficial effects of *Akkermansia muciniphila* and its derivatives on pulmonary fibrosis. Biomed Pharmacother. (2024) 180:117571. doi: 10.1016/j.biopha.2024.117571, PMID: 39418965

[39] Rao Y Kuang Z Li C Guo S Xu Y Zhao D. Gut *Akkermansia Muciniphila* ameliorates metabolic dysfunction-associated fatty liver disease by regulating the metabolism of L-aspartate via gut-liver Axis. Gut Microbes. (2021) 13:1–19. doi: 10.1080/19490976.2021.1927633, PMID: 34030573 PMC8158032

[40] Peng W He CX Li RL Qian D Wang LY Chen WW. *Zanthoxylum bungeanum* amides ameliorates nonalcoholic fatty liver via regulating gut microbiota and activating Ampk/Nrf2 signaling. J Ethnopharmacol. (2024) 318:116848. doi: 10.1016/j.jep.2023.116848, PMID: 37423515

[41] Wu H Lou T Pan M Wei Z Yang X Liu L. Chaihu Guizhi Ganjiang decoction attenuates nonalcoholic steatohepatitis by enhancing intestinal barrier integrity and ameliorating Pparα mediated lipotoxicity. J Ethnopharmacol. (2024) 326:117841. doi: 10.1016/j.jep.2024.117841, PMID: 38310988

[42] Zhang X Coker OO Chu ES Fu K Lau HCH Wang YX. Dietary cholesterol drives fatty liver-associated liver Cancer by modulating gut microbiota and metabolites. Gut. (2021) 70:761–74. doi: 10.1136/gutjnl-2019-319664, PMID: 32694178 PMC7948195

[43] Wang SC Chen YC Chen SJ Lee CH Cheng CM. Alcohol addiction, gut microbiota, and alcoholism treatment: a review. Int J Mol Sci. (2020) 21:6413. doi: 10.3390/ijms21176413, PMID: 32899236 PMC7504034

[44] de Vos WM Tilg H Van Hul M Cani PD. Gut microbiome and health: mechanistic insights. Gut. (2022) 71:1020–32. doi: 10.1136/gutjnl-2021-326789, PMID: 35105664 PMC8995832

[45] Cai J Rimal B Jiang C Chiang JYL Patterson AD. Bile acid metabolism and signaling, the microbiota, and metabolic disease. Pharmacol Ther. (2022) 237:108238. Epub 2022/07/07. doi: 10.1016/j.pharmthera.2022.108238, PMID: 35792223

[46] Parker BJ Wearsch PA Veloo ACM Rodriguez-Palacios A. The genus Alistipes: gut Bacteria with emerging implications to inflammation, Cancer, and mental health. Front Immunol. (2020) 11:906. doi: 10.3389/fimmu.2020.00906, PMID: 32582143 PMC7296073

[47] Martin-Gallausiaux C Marinelli L Blottière HM Larraufie P Lapaque N. SCFA: mechanisms and functional importance in the gut. Proc Nutr Soc. (2021) 80:37–49. doi: 10.1017/s0029665120006916, PMID: 32238208

[48] Yao L. Breaking boundaries: Bacteria act as architects of host T cell modulators using bile acids. Science. (2024) 385:37. doi: 10.1126/science.adq2341, PMID: 38963847

[49] Biagioli M Carino A. Signaling from intestine to the host: how bile acids regulate intestinal and liver immunity. Handb Exp Pharmacol. (2019) 256:95–108. doi: 10.1007/164_2019_225, PMID: 31119464

[50] Jones ML Martoni CJ Ganopolsky JG Labbé A Prakash S. The human microbiome and bile acid metabolism: dysbiosis, dysmetabolism, disease and intervention. Expert Opin Biol Ther. (2014) 14:467–82. doi: 10.1517/14712598.2014.880420, PMID: 24479734

[51] Albillos A de Gottardi A Rescigno M. The gut-liver Axis in liver disease: pathophysiological basis for therapy. J Hepatol. (2020) 72:558–77. doi: 10.1016/j.jhep.2019.10.003, PMID: 31622696

[52] Li J Guo C Liu Y Han B Lv Z Jiang H. Chronic arsenic exposure-provoked biotoxicity involved in liver-microbiota-gut Axis disruption in chickens based on multi-omics technologies. J Adv Res. (2024) 67:373–86. doi: 10.1016/j.jare.2024.01.019, PMID: 38237767 PMC11725159

